# Concerning Operations for Otorrhœa

**Published:** 1908-01-11

**Authors:** 


### Otology.

### CONCERNING OPERATIONS FOR OTORRHŒA.

### III. WITH LATERAL SINUS THROMBOSIS.

Of all the intra-cranial venous sinuses the lateral sinus is that most liable to infective disease, a fact associated with the extreme frequency of middle ear suppuration. The condition is comparable, in a sense, with suppurative phlebitis, say, of the saphenous vein and a neighbouring ulcer on the leg. The dangers of such complications are well known. In the case of the lateral sinus, unless the course of the disease is at once cut short by removal of the septic material from the sinus and its vicinity, the termination is inevitably fatal. Ten to 20 years ago among the first cases of sinus thrombosis submitted to operation, only a few were successfully treated, whereas at the present time, by good management, it is possible to save nearly 70 per cent. (Gower.)

In some cases of chronic otorrhœa the continued inflammation of the bony walls, enclosing the accessory cells of the tympanum, leads to trabecular absorption of the bone. As the process extends backwards it establishes a free communication between the infective contents of the mastoid cavities and the lateral sinus itself by means of cellular connective tissue in large lacunar spaces, through the wall of the sigmoid sulcus. Conveyed along such connective tissue tracks organisms may set up inflammation in the outer layer of the dura mater between the sinus and the bone. With no escape for the products of inflammation, suppuration often ensues, and an extra-dural (perisinous) abscess forms. This is more likely to arise when the plate of bone between the accessory cells and the sinus is unusually thin. Sometimes complete absorption of this plate occurs, so that the sinus is exposed directly to the septic contents of the middle ear. Occasionally acute otitis media induces an infection of the unexposed sinus without any gross lesion in the bone other than that of hyperæmia, due to intense engorgement of the lacunar spaces. As a result of the inflammation of the walls of the sinus the blood adheres as a clot to the inflamed area. As a rule the lumen becomes entirely occluded by clot; this is not, however, invariably the case, and the blood may flow over the diseased area of the sinus, conveying infective material to the heart and lungs in quite the early stages of mural invasion.

In the generality of cases the plastic clot in the sinus increases by direct extension down into the internal jugular vein and backwards towards the torcular Herophili. During this process of extension the original clot where it adheres to the sinus walls rapidly disintegrates and forms what is virtually an abscess within the lateral sinus. Eventually the outer wall of the sinus sloughs. While these changes are going on in the outer wall, the inner wall of the sinus may adhere to the cerebellum, and lead to a cerebellar abscess. In fatal cases the infection is also found to have spread by the venous tributaries to the pia mater and brain cortex. In fact, meningitis or brain abscess is met with in nearly half the fatal cases of lateral sinus thrombosis, and in cases in which the disease has run its course, the neighbouring sinuses may be similarly involved.

Coming to the effects on the general circulation, particles of infective clot may become dislodged in the blood stream and conveyed to the heart or lungs as septic infarcts. The most common manifestations of which are those due to pneumonia and bilateral empyemata. Infective endocarditis and pericarditis are also sometimes met with in fatal forms of the disease.

Passing to the clinical aspects of the disease we find that the general manifestations of lateral sinus infections are toxic in character. As a rule repeated rigors, intermittnt pyrexia, severe sweats, rapid pulse, headache, and vomiting are the preponderating features of the disease. In other cases there is high and continued fever with profuse diarrhœa. The tongue is dry and furred, sordes form on the lips, and the case may suggest typhoid fever. There is, however, a definite leucocytosis, while Widal's reaction is negative.

It is important to realise that sinus infection occasionally presents as a cerebral or meningeal type, even when no brain abscess or lepto-meningitis exists. Such cases may be unjustly marked down as hopeless, whereas they might be saved by timely operation if the true significance of the so-called meningeal symptoms were duly recognised.

Sometimes the pulmonary manifestations predominate, and it is, therefore, the more necessary to be circumspect and to inquire especially for disease of the ear. When otorrhœa is found, or when a history of its occurrence is obtained, the whole mastoid region and side of the neck must be carefully examined. In a large number of cases the local symptoms are well marked. There is definite evidence of middle ear disease, as shown by otoscopy. If there is no mastoid swelling, redness, or tenderness there is often deep-seated pain, and increased sensitiveness to pressure. Sometimes the tenderness is unmistakable, and can be traced along the course of the sinus, towards the occiput. As regards the neck, muscular rigidity, especially of the sterno-mastoid, is commonly found. Glandular enlargement and tenderness in the upper third of the neck along the internal jugular vein are met with when the disease has been in progress some days. Occasionally the whole course of the vein is marked by tenderness.

Evidence of the secondary results of venous obstruction in the circulation of the head and neck are uncommon, but the optic discs may be engorged. Distension of the veins of the face or scalp is only of importance in that it indicates a late stage of the disease. We must bear in mind that it is not a very rare clinical feature of lateral sinus infection to find an almost entire absence of local signs, apart from the otorrhœa. Such cases can only be recognised in the course of a timely operation performed on account of the onset of toxic symptoms in connection with middle-ear suppuration.

*(To be continued.)*

